# Complete Genome Sequence of *Dehalococcoides* *mccartyi* Strain WBC-2, Capable of Anaerobic Reductive Dechlorination of Vinyl Chloride

**DOI:** 10.1128/genomeA.01375-16

**Published:** 2016-12-22

**Authors:** Olivia Molenda, Shuiquan Tang, Elizabeth A. Edwards

**Affiliations:** a Department of Chemical Engineering and Applied Chemistry, University of Toronto, Toronto, Ontario, Canada; b Department of Cell and Systems Biology, University of Toronto, Toronto, Ontario, Canada

## Abstract

*Dehalococcoides mccartyi* strain WBC-2 dechlorinates carcinogen vinyl chloride to ethene in the West Branch Canal Creek (WBC-2) microbial consortium used for bioaugmentation. We assembled and closed the complete genome sequence of this prokaryote using metagenomic sequencing from an enrichment culture.

## GENOME ANNOUNCEMENT

The West Branch Canal Creek microbial consortium (WBC-2) is used for the bioremediation of chlorinated alkenes and alkanes in subsurface environments, most notably 1,1,2,2-tretrachloroethane (1,1,2,2-TeCA) (1, 2). In this consortium, 1,1,2,2-TeCA is dihaloeliminated to 1,2-*trans*-dichloroethene (tDCE) by *Dehalobacter*, tDCE is dechlorinated to vinyl chloride (VC) by *Dehalogenimonas* sp. WBC-2, and VC is dechlorinated to ethene by *Dehalococcoides mccartyi* WBC-2 (2, 3), whose genome is presented here. A subculture enriched solely on tDCE contains only *Dehalogenimonas* sp. WBC-2 (genome available in reference 3) and *Dehalococcoides mccartyi* WBC-2. This subculture has been maintained batch style in 0.5L anaerobic minimal medium with 0.64 mEq tDCE (∼30 mg/L), 3.2 mEq ethanol, and 2.2 mEq lactate since 2010, as previously described (3).

The tDCE subculture DNA was sequenced using a mate-pair large-insert library (8,000-bp insert size, and 100-bp reads with a total 150 million reads) and a paired-end library (150-bp insert size, and 150-bp reads with a total 50 million reads) conducted at the Genome Quebec Innovation Sequencing Centre using Illumina HiSeq 2500. Raw reads were trimmed using Trimmomatic (version 03.0) (4) with default settings to remove adaptors and to filter poor-quality reads. Assemblies were created using ABySS 1.3.7 (5) and ALLPATHS-LG (6) assemblers. Scaffolds produced by ALLPATHS-LG were filled using ABySS contigs, as previously described (3, 7). The finished genome was polished by read-mapping raw reads onto the circular genome using Geneious 8.1.8 (8). All single nucleotide polymorphisms (SNPs) found during read-mapping occurred in fewer than 10% of raw reads mapped to that particular position. The origin of replication was identified using Oriloc (9) in R. The genome was annotated using RAST (8) and BASys (9), followed by manual inspection.

The complete genome is 1.37 Mbp, with 47% G+C content. Based on 16S rRNA identity, this particular *D. mccartyi* belongs to the Pinellas subgroup (10), having 100% 16S rRNA identity with *D. mccartyi* CBDB1 (11) and GT (12). Based on a whole-genome alignment, pairwise identity is 74.6% with CBDB1 and 70.5% with GT. Excluding high-plasticity regions flanking the origin typical of *D. mccartyi* strains (13), the core genome is 94.6% identical to *D. mccartyi* CBDB1. This genome has 15 putative reductive dehalogenase catalytic A subunit (*rdhA*) genes and 12 putative reductive dehalogenase membrane anchor (*rdhB*) genes. *D. mccartyi* WBC-2 contains the previously identified *vcrABC* operon encoding the vinyl chloride reductase (VcrA) located on a genomic island (14). VcrA was the only dehalogenase expressed by this population of *D. mccartyi*, as determined from blue-native polyacrylamide gel electrophoresis coupled with liquid chromatography-tandem mass spectrometry (3). In the current *rdhA* naming system developed by Hug et al. (15), 12 of the 15 *rdhA* genes fall into known ortholog groups (OGs). Two *rdhA* genes have not been previously found in *D. mccartyi*, and one will create a new OG (OG 58), sharing 99.5% amino acid pairwise identity with *D. mccartyi* CBDB1 *rdhA* cbdbA1539. This strain harbors many characteristic features common to *D. mccartyi*, such as an intact prophage, mobile elements, and a multitude of *rdhA* genes whose functions are not yet known.

### Accession number(s).

The complete genome of *Dehalococcoides mccartyi* strain WBC-2 has been deposited in GenBank under GenBank accession no. CP017572.
